# Marijuana promotions on social media: adolescents’ views on prevention strategies

**DOI:** 10.1186/s13011-018-0152-7

**Published:** 2018-07-02

**Authors:** Megan A. Moreno, Aubrey D. Gower, Marina C. Jenkins, Bradley Kerr, Jesse Gritton

**Affiliations:** 1 Department of Pediatrics, University of Wisconsin-Madison, 2870 University Ave, Suite 200, Mailcode 9010, Madison, WI 53705 USA; 2 Seattle Children’s Research Institute, 2001 8th Ave, Seattle, WA 98105 USA

**Keywords:** Marijuana, Policy, Prevention, Adolescent, Youth, Social media, Qualitative

## Abstract

**Background:**

Youth exposure to positive marijuana messages increases their risk of marijuana use. Since Washington State legalized recreational marijuana in 2012, marijuana businesses have used social media business pages to promote their products. Regulations to prevent youth access and targeting by marijuana businesses on social media in Washington State are absent. The purpose of this study was to engage youth in conceptualizing prevention approaches to limit youth exposure to marijuana business promotions on social media.

**Methods:**

Towards our goal of generating novel prevention approaches and promoting youth interaction to build ideas, we used focus groups. Adolescents ages 15-20 years in Washington State were recruited through purposeful sampling to achieve a diverse sample from six schools across two counties. During focus groups, trained facilitators used a semi-structured guide to prompt discussion about marijuana business presence on social media. In the latter half of focus groups, facilitators showed example social media posts from marijuana businesses. All focus groups were audio recorded and manually transcribed. Qualitative analysis was conducted using the constant comparative method.

**Results:**

A total of 32 adolescents with average age 17 years (SD = 0.6), 71% female, 43.8% Asian and 21.9% mixed race, participated in 5 focus groups. Recommendations for prevention focused in two main thematic areas. First, participants supported policies to restrict underage access to marijuana social media pages, an example quote was: “you have access to [the social media page] without being 21 and I think that’s a problem.” Second, participants proposed regulation of content that marijuana companies can post on social media, an example quote was: “I’m thinking they shouldn’t be allowed to use children or anything associated with children and the memes that they post.”

**Conclusions:**

Our findings indicate two strategies to limit youth exposure to marijuana content on social media. These specific strategies represent potential avenues to revise state policies and test the effectiveness of these approaches for states that permit recreational marijuana.

## Background

The emerging landscape surrounding legalized recreational marijuana use presents an optimal moment to consider youth marijuana prevention. In November of 2012, Washington State passed Initiative Measure No. 502 (I-502), which legalized recreational marijuana use for persons over the age of 21. Colorado, Alaska, Oregon, Nevada, Maine, Massachusetts and California are among the additional states that have legalized recreational marijuana use. These laws present new challenges in marijuana prevention for youth.

One challenge is that youth are now presented with increased opportunities for exposure to marijuana messages, including advertisements and promotions. Advertisements may include billboards and print ads; promotions are present on social media sites. As adolescents remain the most ubiquitous and engaged social media users [1], the commercial marijuana business presence in the social media space has high potential to reach and influence youth.

Marijuana companies can create promotional profiles called “business pages” on sites such as Facebook, Instagram and Twitter. These public profiles provide businesses a continuous presence on social media and allow consumers to “follow” their profile. Following a profile establishes an ongoing link between the business and the individual user’s profile, a relationship not possible in traditional broadcast media. When a user follows a business profile, the business can deliver updates and content directly to the user each time the business posts on social media.

Marijuana companies are not required to place age restrictions on access to their profile; thus, youth of any age can access and follow recreational marijuana social media business pages. Followers of the social media profile can respond to posted content by “liking” the content, “sharing” it on their own personal profile and increasing the reach of the content, or by commenting and engaging with the business and others who have commented. Thus, businesses can use social media to attract followers, promote positive marijuana messages (and products) and build an online community using social media. State-based restrictions on marijuana advertising vary in addressing social media promotions; the Washington State Liquor Control Board marijuana social media advertising rules via the Washington Advertising Code (WAC) 314-55-155 do not include any references to social media [2].

Youth online access to retail marijuana companies raises concerns, as previous studies have found that exposure to media messages can influence teens’ perceptions of marijuana as enjoyable or normative. One study surveyed middle school students and found that exposure to advertisements for medical marijuana was associated with stronger intentions to use marijuana [3]. This exposure may have ongoing impact, as another study found that among young adults who had used marijuana, exposure to marijuana advertising was associated with heavier and more potent marijuana use [4].

A further concern is past research demonstrates that marijuana companies overstate the benefits of their products in online settings. A previous study [5] found that 44% of retailers in Washington and 61% of retailers in Colorado promoted health benefits of marijuana consumption on their websites. These proposed benefits included anxiety reduction, depression treatment and insomnia improvement.

Adolescents are a critical population for marijuana prevention efforts. Consequences of marijuana use that are particularly salient to this population include academic difficulties, impaired driving, psychiatric impairment and progression to other drugs [6, 7]. As additional states may legalize recreational marijuana, it is imperative to consider best practices to prevent adolescent marijuana exposure and use. Adolescents have considerable expertise in social media use; thus, incorporation of their views into prevention approaches may help generate relevant strategies [1]. These strategies may inform how states approach social media in their advertising codes. Thus, the purpose of this study was to investigate adolescents’ views regarding prevention of exposure to marijuana message exposure on social media by marijuana businesses in Washington State.

## Methods

This study focused on youth voices and perspectives; which is best suited to a qualitative research design [8]. In order to provide a forum for adolescents to discuss and share opinions, interaction between youth was critical to the study design. Focus groups are a study design in which a group is asked open-ended questions, and communication between research participants is used to generate and process ideas [9].

Focus groups were held between August and December 2016 at community sites and Seattle Children’s Research Institute. The Seattle Children’s Research Institute IRB approved this project. Because of the sensitive nature of the topic to be discussed in a group setting, the IRB placed restrictions on reporting of data with any linked identifiers such as age, gender or personal marijuana use.

### Participants and recruitment

This study was designed to focus on youth ages 15-20 years who currently resided in Washington State. Participant minimum age was selected to be consistent with recommendations for adolescent research participation [10]. Participants with social media experience would be best suited to participate in this study and contribute to discussion. Given that most social media sites limit access to teens age 13 and over, teens aged 15 and over would likely have at least 1-2 years of social media experience. With that experience, they may have seen advertisements and promotions on social media platforms to inform their contributions to the focus group. Participant maximum age was selected to focus on adolescents who were underage for marijuana use and thus would be impacted by any approaches or regulations they proposed. Participants were excluded if they were not English speaking or did not use social media.

Our goal was to recruit a sample to include both community and cultural diversity. First, public high schools and colleges across two counties were identified that represented diversity in race/ethnicity and proportion of students qualifying for free and reduced lunch programs or financial aid to represent socioeconomic diversity. Second, we requested involvement of key contacts at those schools among students who volunteered for this role. Third, key contacts were briefed on the study recruitment goals of diversity and asked to identify potential participants to invite to the focus group. Focus groups were designed to include between 3 and 8 participants, consistent with typical focus group structure. All participants over age 18 provided consent, as did parents of participants under age 18; all adolescents under age 18 provided assent.

### Facilitator guide development and training

The goal of the facilitator guide was to elicit experiences and perspectives to inform prevention strategies. During facilitator guide development, all investigators reviewed the social media promotions pages from marijuana businesses in Washington State on Facebook, Twitter, and Instagram profiles to understand typical posted content. During this review, we identified example posts to share with focus group participants that represented concepts related to youth appeal from previous literature, such as animated characters, candy or sweets, and young-looking people [11, 12]. All investigators reviewed potential example posts via a group discussion, posts with consensus as representing key concepts were included in focus groups.

Two trained facilitators conducted semi-structured focus groups. Facilitator training involved reading focus group training materials, observing standardized focus groups, and conducting a minimum of one previous focus group prior to leading focus groups for this study. Because our research team had previously conducted focus groups on sensitive topics with adolescents, facilitators also reviewed our previous studies using these methods highlighting key approaches for addressing sensitive topics within adolescent focus groups [13–16].

### Focus groups

After the informed consent process was completed, the lead facilitator introduced the project and explained the purpose of the focus group. The lead facilitator explained that the second facilitator would mainly take notes and observe. Lead facilitators were all research staff with college degrees; most of the facilitators had master’s degrees in relevant fields such as public health or education. The lead facilitator highlighted that the focus group would discuss marijuana, a sensitive topic. The focus group participants were told that they did not have to disclose information about their individual experiences with marijuana use, and that all discussions would be recorded and transcribed without identifiers in order to protect their confidentiality.

The facilitator began with open-ended questions about experiences with social media and interacting with businesses on social media. Example questions were: “What are your favorite social media sites to use and why?” and “What do you think about business’ presence on social media?” The discussion was then directed towards sharing experiences regarding viewing marijuana-related content on social media. Example questions included: “Have you ever seen a business page for a marijuana business on social media?” with follow-up questions asking about participants’ views on this content. Participants were also asked: “how do marijuana businesses use social media to promote their products?” After this initial open-ended discussion period, participants were shown several example posts from marijuana businesses on social media and asked to describe their impressions and ideas. An example question was: “What message is this post trying to convey?” Participants were encouraged to discuss their ideas for prevention strategies while viewing these posts.

### Procedures

Focus groups were held in private meeting rooms at the Seattle Children’s Research Institute and lasted between 45 and 65 min. All focus groups were audio recorded and manually transcribed verbatim. In each group, one facilitator recorded written notes and observations during the groups which were included in study data. Participants received a $40 incentive at the end of the focus group.

### Analysis

Three investigators with previous experience in qualitative analysis were involved in the analysis process. The investigators utilized a constant comparative approach. The transcripts were imported to a qualitative analysis program Dedoose (www.dedoose.com). Inductive reasoning based in grounded theory [17] guided codebook development and theme identification.

Prior to analysis, theoretical saturation was evaluated. Theoretical saturation was defined as the phase at which focus group transcripts have been reviewed and it has been determined that no new concepts are emerging. Linkages between the concepts have been noted; supporting that no further data is needed [18]. After completing an initial 3 focus groups, investigators reviewed data and potential themes based on transcripts and written notes. Similarities across groups and emerging themes were noted. Transcript review was again conducted after completion of five groups. At that time, it was determined that based upon the similarities and response across the initial and subsequent groups that theoretical saturation had been reached.

As a first step in the analysis process, 3 investigators reviewed transcripts individually and then met to identify categories to begin the first cycle of data classification. During this meeting, investigators worked collaboratively to develop an initial list of parent and child codes. The parent codes consisted of root codes or overarching categories, while the child codes included sub-categories within the parent codes.

Once consensus was reached on the initial classification criteria, the first cycle of coding began. In this coding cycle, 2 investigators coded one focus group independently, blinded to one another’s data classification. The purpose was to evaluate reliability and validity of the initial classification criteria. After coding this transcript, two investigators met and the focus group transcript was reviewed with codes unblinded to identify discrepancies in coding or categories. The investigators discussed and reached consensus on parent and child code nomenclature, as well as any additions or revisions to coding categories. The coding process was then applied to each subsequent focus group using this constant comparative approach. Throughout this process, the third investigator was available to help reach consensus when the two primary coders were not in agreement.

The second cycle of coding was intended to synthesize and integrate parent and child codes to move towards development of themes and broader concepts. After completion of all coding and review of all coded transcripts by the three investigators involved in analysis, an initial list of themes was developed based on a systematic review of coded excerpts. The thematic content related to recommendations for prevention was then extracted for further examination to ensure clarity and consensus of coders on this content. The investigators met to discuss overlap and discrepancies within codes. After reaching consensus among all 3 investigators, the themes were finalized.

## Results

A total of 32 adolescents participated in 5 focus groups. The average age of participants was 17 years (SD = 0.6), 71% were female. Participants’ race/ethnicity included Asian (43.7%), Multiracial (21.8%) and Caucasian (18.7%). Among participants, 40.1% reported any past use of marijuana, and the average age at first use was 16.1 (SD = 1.4) years. Table 1 shows demographic information from all participants.

**Table 1:** Demographic information for focus group participants

Age	Mean(SD)
	17.625 (1.36)
Gender	n (%)
Male	9 (28.1)
Female	23 (71.9)
Race/ethnicity	n (%)
Asian	14 (43.8)
Mixed/more than one	7 (21.9)
White/Caucasian	5 (15.6)
Black/African American	3 (9.4)
Hispanic	1 (3.1)
American Indian/Alaskan Native	1 (3.1)
Other	1 (3.1)

### Themes

#### Theme 1

Social media sites should block access to marijuana business pages for youth under age 21.

Within all focus groups, participants generated the idea to restrict access to social media business pages for underage youth. A common idea for restriction, sometimes referred to as “age-gating,” was described as limiting access to the social media page based on whether the person was age 21 or over, applying data from their social media profile. Youth described that given that underage people can freely access social media, there needs to be another level of restriction for accessing marijuana business pages. An example comment was “One thing is that I think there should be restrictions on [the marijuana business pages].” Another participant described, “you have access to [the social media page] without being 21 and I think that’s a problem. Like, I don’t think they should be able to put their products directly out there ‘cause anyone can access Twitter.”

In most groups, parallels were drawn to how underage youth could not access social media business pages for tobacco and alcohol due to restrictions on these pages because of age-gating. Youth generally felt that these restrictions for access applied to underage youth were fair and appropriate. Many participants wondered why marijuana companies would be allowed to bypass these age-based restrictions. One participant explained, “I think the idea of comparing it to drinking, and all of the heavy ad laws that are--most people are kind of aware of I feel like--should correspond with marijuana.”

Most participants agreed with the approach of limiting access to business pages, though some expressed pessimism that marijuana companies would follow a rule such as this. This sentiment was often linked to discussions of retail marijuana companies “by definition operating outside the [federal] law.” Other participants expressed a sense of pessimism of whether the rule would be monitored or consequences would happen when it was broken. One participant described, “it seems like, I dunno, there have, there are like implemented laws. But none of them are like really like executed to their word. And uhm, I guess marijuana companies seem, like, they know this and they can do whatever they want. ‘Cause they don’t have any regulation, no one’s actually actively enforcing it. So that’s pretty problematic and maybe uhm if there was enforcement maybe there’d be less of this.”

#### Theme 2

Policy is needed to regulate content that marijuana businesses can use to promote their products.

Many youth participants voiced their thoughts that a policy to restrict access to marijuana business pages alone would “not be enough.” It was recognized that even if youth could not access a marijuana business’s social media page, other social media users with such access could share content from their own profiles and then youth might see it. There were discussions among participants about their experiences seeing branded content promoting marijuana use because it was shared widely on sites such as Twitter, often by peers rather than by a business. One youth noted, “Cause like if the entire purpose of Twitter is to like re-tweet stuff, then if somebody that doesn’t have an age restriction re-tweets it, then you’ve lost all purpose restricting the age.” Another youth stated, “I think in terms of law, that should be an aspect that anything can go viral, so something [about content] should be regulated for sure.”

The discussions about content regulations focused on two key areas: content restrictions and mandated content. Discussions around content restrictions focused mostly around content that would be attractive to youth. One youth summarized it as such: “they shouldn’t direct it towards kids. …I know they shouldn’t be making something that can be harmful to us, attractive to us.” Some participants felt that marijuana social media posts often included people who appeared to be underage, and that this could be misleading to viewers about who was allowed to use marijuana. One adolescent recalled seeing a marijuana business social media post showing a celebrity who she knew was underage using marijuana. She stated, “I think they should make it a law that you shouldn’t post anything about adolescents indulging in illegal substances. They shouldn’t be allowed to post this at all. She is underage and its bad for underage people and it’s misleading.”

Another area of content that participants frequently discussed was marijuana businesses using social media posts to reference popular culture such as celebrities, movies and TV shows. One teen described, “there should be a law saying that you can’t associate marijuana with…movies and stuff like this. Something that is very popular.” Participants felt connected to particular movies, television or celebrities, and felt that integrating popular culture references into marijuana posts was a way to target and engage people their age. One participant described concern about a marijuana business social media post that referenced a TV show she liked. She described, “Um, movies or when you reference movies or popular things on TV shows, then their fandom will also be like ‘Ay, like that’s my favorite TV show.’ And it’s more likely that they will, um look more into your, like, business.”

A few participants suggested that marijuana business should not be able to use memes on their social media pages, given that adolescents and young adults view memes as a popular form of entertainment and communication among their age group. One participant stated “I’m thinking they shouldn’t be allowed to use children or anything associated with children and the memes that they post. This [example] post is so similar to, like, memes that a lot of adolescents engage in.”

Participants also felt that marijuana businesses should not be able to co-brand marijuana with products that they liked, such as sweets, candy or ice cream. One participant explained that linking a non-marijuana product to marijuana “might create associations that shouldn’t be there.” Another participant argued, “I don’t think this should be allowed, to like [brand of ice cream] shouldn’t be allowed. To advertise this, and that, like, [brand of ice cream] is a family establishment for all ages. And not all ages are, like, able or supposed to be purchasing pot.”

Some participants argued that businesses should be limited in their ability to make claims about health benefits of marijuana use. One participant explained that rules should be present “where they can’t like per- like promote like false information about h- health benefits of tobacco, like kind of similar to that.” Another suggestion was that for every post promoting benefits of marijuana use, the company should have one post about health risks of marijuana use. One participant remembered learning about this approach in school, “I’m not really aware of advertising laws specifically, but I can recall from class being told that for every cigarette ad there was on TV, there had to be another ad that followed it saying…talking about the negative impact of smoking cigarettes.” This idea resonated with several participants, with endorsements including, “I would just follow up with what she was saying and say that with the whole smoking cigarettes, and how you see that with advertisements, that with every pro they need to follow up with the cons.”

Additional ideas focused on content that should be present or mandated on marijuana business social media sites as well as their posts. For example, to require labeling of social media content as being only appropriate for those over age 21. Others confirmed this should happen even if social media sites were restricted. One participant explained, “I feel like they should probably put on the actual advertisements for actual businesses. Like, 21 or the age that it is actually for, instead of just being put out for just everyone. Even if it’s, like, obvious that they can screen for the younger people, it should be, like, said.”

A few participants argued that disclaimers about health risks or consumption limits should be present on business’ social media posts about marijuana. One participant described, “I think that more knowledge about the possible bad effects of it [should be posted]. Like, I didn’t know it was bad for a long time.” Participants again referenced alcohol and tobacco approaches and suggested applying these strategies to marijuana would be consistent with what they had seen in other media such as movies. One example was described, “in movies or something when they’re showing, like, alcohol or something. And, like, the little disclaimer on the bottom they write. Like, alcohol use can be injurious to health.” However, some participants were pessimistic that companies could be persuaded to post this type of content, as one participant described, “I think my last thing is just, like, [marijuana] businesses could care less about the health aspect.”

Some participants felt that marijuana social media posts should provide education about safe use. One participant stated, “I also feel like they should, like, notify people of the risk if there is any. And, like, what is too much or like how…what’s the limit that one should have or you know, like, with alcohol you know.” These discussions often drew parallels to how alcohol messages often include descriptions of blood alcohol limits for driving.

## Discussion

This qualitative focus group study engaged diverse youth to elicit their views and recommendations for preventing youth access to marijuana business messages via social media. We found that participants were able to identify several aspects of marijuana business social media posts that had youth appeal. Further, the majority of participants supported policies to regulate marijuana business social media pages, and these policy ideas generally centered on restricting both access and content.

A first important finding from this qualitative study is that participants focused on policy as a way to prevent adolescent exposure to marijuana promotions. Approaches involving community, school, family or individual prevention strategies were notably absent from the focus group discussions. The focus on policy may have been rooted in previous experiences; most participants mentioned learning about alcohol and tobacco regulations and proposed similar strategies for marijuana. The ties to alcohol and tobacco are highly salient, as the scientific literature is replete with studies showing how these companies have used youth-friendly messages and images to entice underage adolescents, and the effectiveness of these approaches. For example, previous studies have illustrated that adolescents’ responses to cigarette ads can change the appeal of smoking [19, 20] and may be able to recruit new adolescent smokers [21–23]. A recent systematic review summarized numerous studies showing associations between exposure to alcohol advertising and youth drinking behavior [24]. Adolescents’ recommendations indicate an understanding of these critical links between message exposure and behavior; teens were willing to propose and support restrictions on marijuana companies’ ability to target their age group.

In interpreting this finding, it is important to note that the recommendations by youth participants in this study were similar to an early version of the WAC describing marijuana business messaging regulations: *“Please use social media with caution and be mindful not to appeal to, or solicit, viewers under the age of 21. If possible, please restrict views to adults age 21 and older*” [2]. This WAC was revised in 2015 to remove the social media specific content.

A second finding is participants’ insight regarding content that was perceived as appealing to their age group. This type of content included references to popular culture such as movies and memes, co-branding marijuana with products such as sweets, as well as inaccurate content promoting marijuana as a way to improve health. It is interesting that there was substantial overlap in the proposed content restrictions by participants and the types of restrictions on tobacco and alcohol marketing, such as limiting cartoons like the infamous “Joe Camel” [25, 26].

One critical difference to recognize in the marijuana social media business approach compared to traditional advertisements is that companies can take appealing content created by other companies, such as a movie quote or a meme, and post it on social media to promote their own company and marijuana product. The culture of social media allows for sharing content created by others, and this provides nearly limitless access to popular movies, television, celebrities, and products that appeal to youth as a source of content to post on social media. The ability to share existing and popular content via social media represents a new approach to influence youth that merits further study. Another difference is that marijuana companies can co-brand their product with other child-friendly products, as was noted by participants in the example post linking the marijuana business to a local brand of ice cream. As participants pointed out, this linking of products could create an association between a beloved youth-friendly product and marijuana.

Limitations to this study include the geographic focus of our study, which was intentional given Washington State recreational marijuana laws and our qualitative design, but limits generalizability. Our study utilized a purposeful sample focused on recruitment of diverse youth, though our sample was not random nor generalizable by design. Because of the qualitative study design, we cannot comment on adolescents who were approached to participate and declined. The use of key contacts may have led to key contact identifying students who were similar to them in views or perspectives, because we did not include pre-screening processes among those invited to participate we cannot verify if this was the case. Key contacts were encouraged to identify students from different classes, organizations or peer groups. The themes in this study were generated through investigator driven analysis, and may not represent themes that a youth would derive from the data. Because this study involved youth and marijuana, the IRB placed protections on how we represented the data. We are unable to add participant numbers or identifiable characteristics for quotations. Though this was not evaluated in our study, the influence of personal use of marijuana on interpretation of social media content, as well as the impact on the teens themselves who view marijuana social media content is worthy of future study.

## Conclusion

Alcohol and tobacco industry advertising guidelines include provisions restricting access and specific content. There are no such industry codes for marijuana companies. As marijuana advertising and promotions are still in their infancy, understanding emergent issues that have arisen while only a few states have legalized marijuana may help inform policies that prevent future harm. Our findings bring youth voices to support the development and implementation of policies to restrict underage access to and regulation of marijuana businesses’ content on social media.

Based on these findings, we recommend that all states with legalized recreational marijuana should implement specific policies related to social media to restrict youth access and youth-friendly content. These policies should include language to implement requirements for all marijuana businesses to use age-gating (i.e. limiting page access by age present on the user’s profile) on social media. The prevalence and effectiveness of marijuana business page age-gating has not been assessed, though studies of age-gating to prevent youth access to alcohol business pages has shown poor compliance by alcohol companies [27]. Thus, routine evaluations of age-gating needs to be part of the implementation and ongoing monitoring of these rules.

Further, we recommend that content appealing to youth should be restricted and all social media posts must include warnings. The content restrictions can be modeled off advertisements in the alcohol and tobacco realm, as well as data collected in this study pertaining to social media memes and promotion of linked products.

The routine monitoring and implementation of these rules clearly presents challenges to state organizations who are charged with developing and implementing these restrictions. Leveraging fines for non-compliance in this multimillion dollar industry may offset the costs to state agencies tasked with monitoring social media, a continuously moving target. Thus, research on strategies to monitor and identify infractions is needed. Another possible avenue is to work directly with marijuana businesses to promote best practice sharing and collaboration to ensure compliance. Given the importance of youth marijuana prevention, policies to prevent youth access to influential marijuana messages on social media are a critical consideration for lawmakers, prevention scientists and child advocates.
